# Anamnestic risk factor questionnaire as reliable diagnostic instrument for osteoporosis (reduced bone morphogenic density)

**DOI:** 10.1186/1471-2474-12-187

**Published:** 2011-08-17

**Authors:** Leila Kolios, Caner Takur, Arash Moghaddam, Mirjam Hitzler, Heinrich Schmidt-Gayk, Arnold J Suda, Bernd Höner, Paul A Grützner, Christoph Wölfl

**Affiliations:** 1 Department for Plastic-, Reconstructive and Handsurgery, Burn Care Centre, BG Unfallklinik Ludwigshafen, Ludwig-Guttmann-Str.13, 67071 Ludwigshafen, Germany; 2 Department of Trauma and Orthopaedic Surgery, BG Trauma Center Ludwigshafen, Ludwig-Guttmann-Str.13, 67071 Ludwigshafen, Germany; 3 Clinical Laboratory Limbach, Im Breitspiel 15, 69126 Heidelberg, Germany; 4 Department of Social and Legal Sciences, SRH Hochschule Heidelberg, Ludwig-Guttmann-Str.6, 69123 Heidelberg, Germany

## Abstract

**Background:**

Osteoporosis is a major health problem worldwide, and is included in the WHO list of the top 10 major diseases. However, it is often undiagnosed until the first fracture occurs, due to inadequate patient education and lack of insurance coverage for screening tests. Anamnestic risk factors like positive family anamnesis or early menopause are assumed to correlate with reduced BMD.

**Methods:**

In our study of 78 patients with metaphyseal long bone fractures, we searched for a correlation between anamnestic risk factors, bone specific laboratory values, and the bone morphogenic density (BMD). Each indicator was examined as a possible diagnostic instrument for osteoporosis. The secondary aim of this study was to demonstrate the high prevalence of osteoporosis in patients with metaphyseal fractures.

**Results:**

76.9% of our fracture patients had decreased bone density and 43.6% showed manifest osteoporosis in DXA (densitometry) measurements. Our questionnaire, identifying anamnestic risk factors, correlated highly significantly (p = 0.01) with reduced BMD, whereas seven bone-specific laboratory values (p = 0.046) correlated significantly.

**Conclusions:**

Anamnestic risk factors correlate with pathological BMD. The medical questionnaire used in this study would therefore function as a cost-effective primary diagnostic instrument for identification of osteoporosis patients.

## Background

The WHO included osteoporosis in the list of the ten major worldwide diseases [1]. Approximately 200 million patients worldwide, 44 million in the US and about 7.6 million in Germany, suffer from osteoporosis [2-4]. The high prevalence of osteoporosis in current populations becomes more stressing to health care systems as osteoporosis rates increase with age, and as populations adopt unhealthy lifestyles with reduced physical activity and unbalanced diets [5]. In the western industrial nations there is an immense imbalance between inadequately treated osteoporosis and the continually increasing life expectancies [6]. In Germany, costs incurred due to osteoporosis result from osteoporosis associated fractures, especially fractures of proximal femur, and include acute care, rehabilitation, and extended nursing care. All in all, the expenditure amounts to 5 billion euros annually [6,7]. A proximal femur fracture is often the first indication of manifest osteoporosis. Preventional diagnostics such as DXA-measuring or laboratory examination, amounting to approximately 15 million euros per year, are not currently covered by health insurance.

There are various risk factors, which are assumed to highly correlate with osteoporosis. These are anamnestic details of the patient's medical history as for example: positive family anamnesis, early menopause, nicotine abuse, age > 70 years. They are included as osteoporosis-specific risk factors in the recommendations of the German Society of Osteology (DVO, Dachverband Osteologie e.V.). The aim of this study is to demonstrate the high prevalence of osteoporosis in patients with metaphyseal fractures, and, secondly, to show the competence of anamnestic risk factors and bone specific laboratory values in osteoporosis identification.

## Methods

Between January 2008 and May 2010, 78 patients aged 40 to 80 years who presented to our hospital with a metaphyseal fracture of the distal radius, proximal humerus and proximal femur with an obligatory indication for surgical stabilization were included in this study. Exclusion criteria were: polytrauma, significant soft tissue injury, extensive open fractures, > 24 h mechanical ventilation after surgery, dialysis, collagenosis, chronic inflammatory bowel disease, haematological disorders and malignancies, and long-term therapy with immunosuppressants.

X-rays of the fracture region and the lumbar spine in anterior-posterior and lateral views were performed of all patients. Lumbar spine radiographs were used to exclude morphological changes of the vertebrae leading to inaccurate values in DXA bone density measurement. BMD was examined at the lumbar spine, total hip and hip subregions using dual energy X-ray absorptiometry DXA (Lunar iDPX, GE Medical Systems Germany, Solingen, Germany) based on Encore TM Version II.X software.

Using normative data for young Caucasian adults, BMD was categorized as normal, low, or osteoporotic, as defined by the World Health Organization [8]. Participants with a T-score ≤ -2.5 SD were categorized as having osteoporosis.

To collect the anamnestic risk factors of patients, we established a standardized questionnaire according to the recommendations of the German Society of Osteology (DVO, Dachverband Osteologie e.V.). The questionnaire included clinical risk factors (19 for men, 21 for women) as shown in table 1 [9]. The questionnaire was filled out with one member of the study group present, to provide assistance if needed.

**Table 1:** Medical questionnaire of anamnestic risk factors for reduced BMD

	men	women	total
**Reduction of body height > 4 cm**	3	9	12

**Alcohole abuse**	2	4	6

**Age > 70**	2	14	16

**Anorexia nervosa**	0	0	0

**Anticonvulsive therapy**	1	0	1

**Already suffered osteoporotic fractures**	2	9	11

**BMI < 20 kg/m^2^**	0	0	0

**Chronical renal or hepatic disease**	0	2	2

**Family history**	3	19	22

**Early menopause (< 45. Lj.)**	-	13	13

**Late menarche (> 15. Lj.)**	-	2	2

**Highdose heparintherapy**	0	1	1

**Hormon replacement therapy**	0	6	6

**Hyperparathyroidism**	0	0	0

**Hyperthyroidism**	0	4	4

**(N)IDDM**	1	3	4

**Immobilisation, Inactivity**	1	1	2

**Malabsorption-syndrome**	0	0	0

**Multiple Sclerosis**	0	1	1

**Nikotine abuse**	3	12	15

**Rheumatoid Arthritis**	0	4	4

The right columns show the frequence of aberrations of the single risk factors in our study.

Laboratory analysis was performed the morning after injury in a fasting state. Eleven bone specific parameters reflecting bone metabolism were examined: intact PTH [10], 25-hydroxy vitamin D3 (25-OH-D3) [11], beta-crosslaps (ß-CTX), pyridinoline in urine, desoxypyridinoline in urine (DPD) [10,12], procollagen type 1 N propeptide (PINP) [10], creatinine in urine [13], estradiol [10], homocysteine, vitamin B12 and folate [14,15]. Every pathologic aberration from the standard value was assessed with one point.

Statistics were performed using SPSS 11.0.0 (IBM Germany, Munich, Germany) and Microsoft Excel 2003/2007 software, Microsoft Corp. Washington, USA. Using Spearman's rank correlation coefficient, the single anamnestic risk factors and laboratory parameters were correlated with the reduced BMD values. P ≤ 0.05 was considered to be significant and p ≤ 0.01 highly significant. In addition we performed a multivariable analysis in order to adjust for potential confounders. With the method of "ordinal logistic regression" we examined age, sex and BMI as possible confounders with the variables BMD, risk factors and laboratory values.

The study is approved by the local Ethical Committee of Heidelberg with the approval number 1572002. All patients gave their informed consent prior to their inclusion in the study.

## Results

A total of 78 patients who suffered a metaphyseal fracture of the distal radius, proximal humerus or proximal femur between January 2008 and May 2010 were included in this study. The average patient age was 62.2 years with a range from 41.9 to 77.6 years. The 12 male patients had an average age of 65.4 years, the 66 female patients of 61.6 years. 41 patients had a distal radius fracture, 23 patients a proximal humerus fracture and 14 patients suffered a proximal femur fracture.

**Figure 1:**
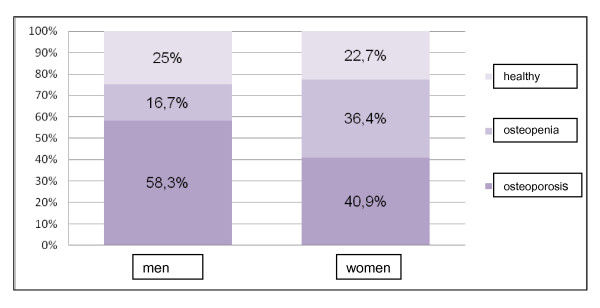
**Results of DXA-measuring**. Percentaged amount of healthy, osteopenic and osteoporotic values in women and men.

In DXA-measuring 76.9% of patients showed a pathologic bone density. 34 (43.6%) patients had a decrease in bone density in terms of osteoporosis, 26 (33.3) patients had osteopenic BMD. 18 (23.1%) of patients had physiological findings (Figure 1). Among the male patients, 58.3% showed manifest osteoporosis and 16.7% osteopenia. Female patients showed in 40.9% manifest osteoporosis and in 36.4% an osteopenia.

The frequency of the single anamnestic risk factors can be seen in table 1. The leading risk factors are: positive family history, age > 70 years, nicotine abuse, early menopause, reduction of body height > 4 cm and previous osteoporotic fractures. There was no incident of 4 risk factors (anorexia nervosa, BMI < 20 kg/m^2^, hyperparathyreoidism, malabsorption syndrome) in our collective.

**Figure 2:**
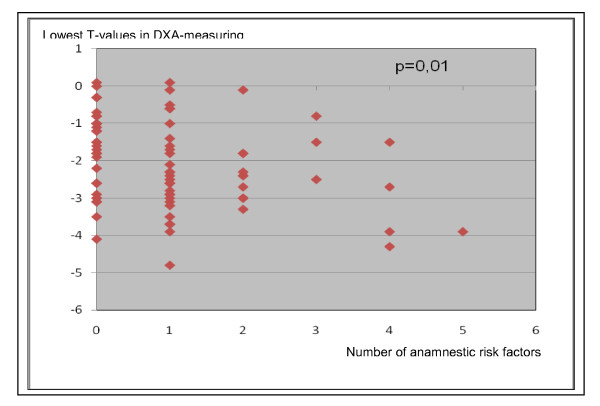
**Diagram presenting the correlation between the lowest T-values of DXA-measuring with increasing number of anamnestic risk factors**. The correlation of the most important risk factors and a reduced BMD is highly significant (p = 0.01, Spearman's rank correlation).

Questionnaire risk factors correlated with reduced bone density values. Spearman's rank correlation allowed separate consideration of the individual risk factors reduced bone density. The 10 anamnestic risk factors: reduce of body height > 4 cm, alcohol abuse, age > 70 years, anticonvulsant therapy, previous osteoporotic fractures, early menopause (< 45years), hyperthyroidism, immobilization, multiple sclerosis and nicotine abuse showed a highly significant correlation (p = 0.01) with low bone density (Figure 2).

Table 2 shows the frequency of pathological laboratory values in our collective. Laboratory values of 67 patients were evaluated; 11 patients declined blood sample collection. The main pathologic laboratory values are: homocysteine (51 patients), 25-OH-D3 (42 patients), and desoxypyridinoline (31 Patients). The fewest aberrations were seen in ß-CTX (13 patients), estradiol (7 patients) and vitamin B12 (6 Patients) measurings.

**Table 2:** Frequence of aberrations of bone specific laboratory parameters in women, men and in total

	men	women	total
**Parathormone intakt**	5	22	27

**25 (OH) D3**	7	35	42

**ß-CTX**	2	11	13

**Pyridinoline (urine)**	4	25	29

**Procollagen Typ I N propeptide**	1	14	15

**Desoxypyridinoline (urine)**	4	27	31

**Creatinine (urine)**	6	12	18

**Estradiol**	1	6	7

**Homocysteine**	7	44	51

**Vitamin B12**	2	4	6

**Folate**	3	22	25

**Figure 3:**
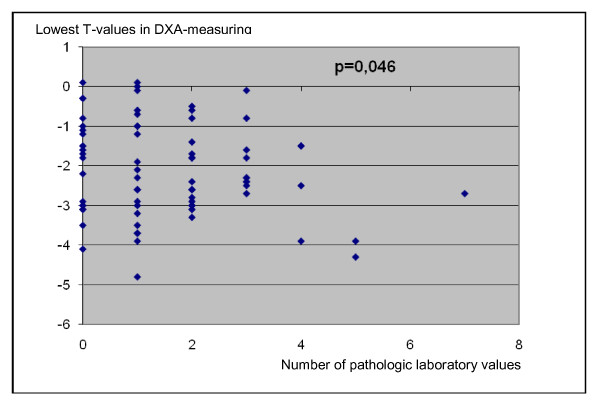
**Diagram presenting the correlation between the lowest T-values of DXA-measuring with increasing number of pathologic laboratory parameters**. The correlation of the seven bone specific values and a reduced BMD is significant (p = 0.046, Spearman's rank correlation).

For statistical analyses, Spearman's rank was used to correlate abnormal laboratory values with a decrease in bone density. The highest correlation (p = 0.046) was noted in parathyroid hormone, 25-OH-D3, procollagen type 1 N propeptide, desoxypyridinoline, creatinine, homocysteine and vitamin B12 (Figure 3).

The multivariable analysis showed no significant effects with the variables; thus the age, sex and BMI of patient do not have a confounding effect.

## Discussion

In this study, nearly 80% of patients who suffered a metaphyseal fracture showed pathologically reduced bone mineral density, and nearly 50% of the patients were diagnosed with manifest osteoporosis. 84.6% of our patients were postmenopausal women. Our results coincide with current research; Vogel et al. [16] found osteoporosis in 72% of acute limb fracture patients (only 4% showed normal bone density). Another Dutch study [17] evaluated fractures resulting from low energy trauma and found manifest osteoporosis in 67% of patients. (Hegemann et al defined the T-Score as < -2.0 for osteoporosis, compared to < -2.5 in our study).

Having identified one of the major problems in treatment of osteoporosis: post fracture diagnosis; we have identified the major cause of the progression of this disease: the therapeutic window in which to minimize fracture risk is overlooked. Due to the exceptionally high morbidity and mortality after osteoporotic fractures, prevention is essential. However, as mentioned, the costs for preventional screenings such as DXA-measurement or laboratory examination (amounting to approximately 15 million euros per year) are not currently covered by German health insurance companies [6]. Furthermore, bone loss progresses silently, and without patient education and preventional screenings, few patients would recognize the risk [18,19].

A safe, simple and cost effective instrument for osteoporosis diagnosis is essential. The results of this study show that a simple medical questionnaire containing 10 anamnestic questions is a reliable test to quickly and efficiently screen large numbers of patients for reduced BMD. With early detection of bone loss and consecutive implementation of antiosteoporotic therapy, the risk of osteoporotic fractures with the coinciding high morbidity and mortality can be reduced, thereby achieving a massive decrease in health care costs.

The most important risk factors identified by the questionnaire are as follows:

Patient age represents a core variable for osteoporosis risk [20] increasing age increases fracture risk [8,21]. Age related decreases in estrogen lead to disrupted microarchitecture of trabecular bone [22]. Thus, age is one of the most important risk factors for osteoporosis and therefore was included in the DVO.

The loss of ≥ 4 cm body height is risk factor for reduced BMD, and may result from vertebral sintering due to osteoporotic change of trabecular structure. Joakimsen et al. [23] showed that patients with a maximal height of 168 cm suffered fracture of the lower extremity 2.5 times more often.

Excessive alcohol consumption showed high significance with reduced BMD in our study. Kanis et al. [24] also demonstrated a correlation between high amounts of alcohol consumption and reduced BMD. The cause may be increased renal secretion of calcium. On the other hand, low alcohol consumption (one glass of wine per day) can positively influence bone mineral density [25].

Similar osteoporotic findings exist for nicotine abusers, due to a reduced enteral resorption of calcium [26]. Comparing smokers and non-smokers Kanis et al. [24] showed a significantly increased fracture rate in smokers. In addition, Law et al. [27] found out that the risk of bone loss caused by smoking is especially high in postmenopausal women over the age of 60.

In the past, the high fracture rate under epileptic patients was explained by the convulsive seizures; however, the anticonvulsive pharmaceuticals are now known to reduce the enteral resorption of calcium, and thereby causing the high prevalence of reduced bone density found in this population [28-30].

Early menopause, before age 45, is also defined as a risk factor for decreasing bone mass. The comparatively short estrogen exposition time is thought to enhance bone resorption [31]. Other authors propose nutrition as the cause [32].

Recent studies show that subclinical hyperthyroidism leads to a decreased bone mineral density [33,34]. Postmenopausal women with hyperthyroidism are especially vulnerable to bone mass loss [35]. The mechanism of action has been proposed by Katani et al. [36] and [37] Garnero et al. to be trijodthyronine (T3) induction of differentiation of osteoclasts. This leads to the creation of bone resorption products (e.g. pyridinoline, peptides of collagen) and excretion of calcium in the urine. T3 also stimulates osteoblasts, but all in all the balance is negative.

Another highly significant factor in our study was the immobilisation or inactivity of patients. Also Dargent-Moline [38] showed that a reduced mobility, activity and reduced musculature increases the risk of developing osteoporosis. Due to the missing mechanical exposure of the muscular-skeletal system, the bone tissue weakens [39]. This explains why patients with multiple sclerosis suffer a significantly reduced bone mineral density [40].

Our questionnaire represents a screening method; if reduced BMD is suggested, a complete diagnosis with clinical examination, laboratory tests, radiography and DXA measurement remain the gold standard to achieve a precise diagnosis of the extent, cause, and indicated treatment of pathological bone density.

As for the bone specific laboratory values, out of the 11 factors examined in this study, 7 showed a significant correlation to a reduced BMD, and are discussed below.

Most of our patients showed a lack of 25-OH vitamin D3, probably due to the reduced exposure to sunlight during winter. The lack of D3 leads to PTH increase which induces calcium decomposition in bone; possibly the cause of the high PTH levels found in our patients. Another cause of elevated PTH is primary or secondary hyperthyroidism [41-43]. Both low D3 and high PTH are important parameters in the diagnosis and treatment of osteoporosis in the DVO guidelines [9].

One of the most significant markers for osteoblast activity is the PINP (procollagen type 1 N propeptide), which is separated during synthesis of collagen. Increased values indicate active bone formation, and is typical for malignant tumors or osteoporosis [12,44,45].

Desoxypyridinoline is a decomposition product of collagene and represents a specific marker for osteoclast activity. Various studies proved its reliability [46-48].

Creatinine levels detect secondary osteoporosis due to renal osteopathy. Homocysteine is mainly used for the identification of an atherosclerotic risk profile. Increased blood levels of homocysteine in conjunction with decreased vitamin B12, leads to a reduced BMD and, as reported by Leboff et al. [49] results in osteoporotic fractures. Further studies presented same findings [50,51]. Stimulation of osteoclast activity is presumed to be the mechanism by which a high level of homocysteine and a low level of vitamin B12 reduces of BMD [52].

## Conclusions

In conclusion, there is a high prevalence of undiagnosed osteopenia and osteoporosis in patients suffering metaphyseal fractures. Both methods (medical questionnaire and laboratory analysis) examined in our study present reliable instruments for diagnostics of osteoporosis. Astonishingly, the cost-effective and "simple" medical questionnaire showed a high correlation with reduced BMD and therewith presents next to the laboratory values a powerful instrument for osteoporosis diagnostics.

### Integrity of research and reporting

The study is approved by the local Ethical Committee of Heidelberg with the approval number 1572002. All patients gave their informed consent prior to their inclusion in the study.

## Competing interests

The authors declare that they have no competing interests.

## Authors' contributions

LK participated in the evaluation of patients, data, statistics and wrote the paper, she has seen and approved the final version. CT participated in planning of the study, evaluation of patients and data and has seen and approved the final version. AM participated in planning of the study, evaluation of patients, data and statistics and has seen and approved the final version. MH participated in planning of the study, evaluation of patients and data and has seen and approved the final version. AS participated in planning of the study, evaluation of patients, data and statistics and has seen and approved the final version. HS-G participated essentially in establishment of laboratory analyses, their evaluation and statistics, but unfortunately died before submission of this study. BH participated substancially in planning of the study and performing of statistics and has seen and approved the final version. PAG participated in planning of the study, supervision of the study, evaluation of patients, data and statistics and has seen and approved the final version. CW participated substancially in planning of the study, supervision of the study, evaluation of patients, data and statistics and has seen and approved the final version.

## Pre-publication history

The pre-publication history for this paper can be accessed here:

http://www.biomedcentral.com/1471-2474/12/187/prepub
